# Outcomes of Patients with Active Cancers and Pre-Existing Cardiovascular Diseases Infected with SARS-CoV-2

**DOI:** 10.21203/rs.3.rs-2952641/v1

**Published:** 2023-05-23

**Authors:** Brijesh Patel, Scott Chapman, Jake Neumann, Aayush Visaria, Oluwabunmi Ogungbe, Sijin Wen, Maryam Khodaverdi, Priyal Makwana, Jasvinder A. Singh, George Sokos

**Affiliations:** West Virginia University; University of Minnesota College of Pharmacy; West Virginia School of Osteopathic Medicine; Rutgers Robert Wood Johnson Medical School; Johns Hopkins University; West Virginia Clinical and Transitional Science Institute; West Virginia Clinical and Transitional Science Institute; West Virginia Clinical and Transitional Science Institute; University of Alabama at Birmingham (UAB); West Virginia University

## Abstract

**Objective:**

To determine the impact of acute SARS-CoV-2 infection on patient with concomitant active cancer and CVD.

**Methods:**

The researchers extracted and analyzed data from the National COVID Cohort Collaborative (N3C) database between January 1, 2020, and July 22, 2022. They included only patients with acute SARS-CoV-2 infection, defined as a positive test by PCR 21 days before and 5 days after the day of index hospitalization. Active cancers were defined as last cancer drug administered within 30 days of index admission. The “Cardioonc” group consisted of patients with CVD and active cancers. The cohort was divided into four groups: (1) CVD (−), (2) CVD (+), (3) Cardioonc (−), and (4) Cardioonc (+), where (−) or (+) denotes acute SARS-CoV-2 infection status. The primary outcome of the study was major adverse cardiovascular events (MACE), including acute stroke, acute heart failure, myocardial infarction, or all-cause mortality. The researchers analyzed the outcomes by different phases of the pandemic and performed competing-risk analysis for other MACE components and death as a competing event.

**Results:**

The study analyzed 418,306 patients, of which 74%, 10%, 15.7%, and 0.3% had CVD (−), CVD (+), Cardioonc (−), and Cardioonc (+), respectively. The Cardioonc (+) group had the highest MACE events in all four phases of the pandemic. Compared to CVD (−), the Cardioonc (+) group had an odds ratio of 1.66 for MACE. However, during the Omicron era, there was a statistically significant increased risk for MACE in the Cardioonc (+) group compared to CVD (−). Competing risk analysis showed that all-cause mortality was significantly higher in the Cardioonc (+) group and limited other MACE events from occurring. When the researchers identified specific cancer types, patients with colon cancer had higher MACE.

**Conclusion:**

In conclusion, the study found that patients with both CVD and active cancer suffered relatively worse outcomes when they had acute SARS-CoV-2 infection during early and alpha surges in the United States. These findings highlight the need for improved management strategies and further research to better understand the impact of the virus on vulnerable populations during the COVID-19 pandemic.

## Introduction

Since the beginning of the SARS-CoV-2 pandemic in 2019, more than 625 million cases have been confirmed worldwide, and more than 6 million people have died from a SARS-CoV-2 infection and associated complications; more specifically in the United States, over 95 million cases have been confirmed and more than 1 million deaths have been reported (https://covid19.who.int/ [accessed 10.31.22]). Individuals with select comorbidities and from certain populations have been disproportionately affected by SARS-CoV-2 infection and associated complications.^1,2^ For example, patients with cancers are considered at greater risk to have poorer outcomes from SARS-CoV-2-related sequelae due to their immunocompromised state associated with chemotherapy, which suppresses the immune system and increases their risk for poor outcomes.^3^ Similarly, cardiovascular disease (CVD) is an important risk factor for mortality in patients infected with SARS-CoV-2.^4^ Patients with lower cardiac reserve, increased susceptibility to arrhythmia, and the inability to augment cardiac output in response to increased stress during an acute illness event could result in poor outcomes.

The risk of severe SARS-CoV-2 infection could be greater in patients with coexisting cancer and CVD, leading, lead to poorer outcomes. In a retrospective study conducted at a single healthcare system, patients with cancer and CVD were at an increased mortality risk than those with either condition alone.^5^ A SARS-CoV-2 cardiovascular disease registry showed that the presence of CVD risk factors itself was not a predictor of mortality in patients with cancer, likely due to significant interaction with CVD and cancer risk factors.^6^ The major limitations of these studies were that they were done in the early phase of the SARS-CoV-2 pandemic when optimal management strategies were evolving. Therefore, we sought to investigate the SARS-CoV-2 infection outcomes inpatients with concomitant active cancer and CVD. We conducted a large, retrospective, registry-based study using data from the National COVID Cohort Collaborative (N3C) supported by National Center for Advancing Translational Sciences of the National Institute of Health (NIH).

## Methods

### Data Source

The N3C is an open science community database focused on analyzing patient level Electronic Health Record (EHR) data harmonized from 72 sites across the US.^7^ Briefly, the N3C includes inpatient and outpatient cases after January 1, 2020, with the potential for data at the hospital site before the case up to January 1, 2018. Sites contributing patient-level data from the EMR are transferred to N3C, where the data is received and undergoes several steps to harmonize from into OMOP concepts and perform quality checks. A data use agreement with the University of West Virginia and N3C allows for access to the de-identified data in the N3C data Enclave. The research was reviewed by the UWV IRB (Protocol# 2101218288) and granted exemption status. All authors who performed analyses and had access to N3C data in the Enclave obtained individual institutional review board approvals from their respective institutions for this project and were also approved to use a limited data set. N3C approved the research as limited data set (level 3) by the N3C Data Use Request Committee, allowing access to actual dates of patient care interventions.

## Patients, data, and outcomes

Patients in the N3C database were identified by searching for patients captured in predetermined concept sets created using the International Classification of Diseases, tenth revision (ICD-10) codes, RxNorm, and LOINC codes on the N3C platform. Patients with a history of CVD and a diagnosis of an active oncologic condition, and whether they were confirmed to have a positive (or negative) SARS-CoV-2 infection between January 1, 2020 and July 21, 2022, defined as RT-PCR positive (or negative) test for SARS-CoV-2 within 21 days before or 5 days after the index admission. Cancers that were excluded included non-melanoma skin cancers, benign tumors, or unknown types. We defined patients with active cancer if they received chemotherapy within 30 days before or after the index admission. Four cohorts of patient groups were defined: 1) Patients with CVD but SARS-CoV-2 negative (CVD (−)), (2) Patients with CVD and active cancer but SARS-CoV-2 negative (Cardioonc (−)), (3) CVD and SARS-CoV-2 positive (CVD (+)), and (4) CVD and active cancer and SARS-CoV-2 positive (Cardioonc (+)). The concept sets also collected patient demographics, characteristics, comorbidities, and antineoplastic agents. The primary outcome of this analysis was major adverse cardiac events (MACE), composed of acute heart failure, stroke, myocardial infarction, and death as all-cause mortality. The outcomes of interest were evaluated over time for each cohort. Additional sub-group analyses of the outcomes were conducted based on the cancer type, for lung, breast, prostate, colorectal, and hematological cancers separately. To account for differences in the outcome as treatments became available and care approaches evolved, we also sub-grouped the comparison of patient outcomes into categories of SARS-CoV-2 variants (early, alpha, delta, and omicron) by each period of time when different variants of SARS-CoV-2 were predominant in the US. We defined Early SARS-CoV-2 from January 1, 2020 to February 13, 2021; Alpha from February 14, 2021 to July 6, 2021; Delta from July 7, 2021 to January 08, 2022 and Omicron January 09, 2022 to July 21, 2022 (last date of inclusion). We created the specific date cutoffs based on the prevalence of waning strain no more than 10% (https://nextstrain.org/)

### Statistical Analysis:

We summarized patient characteristics using descriptive statistics such median and quartile or means and standard deviations, proportions, and included summary tables, bar-plots, box-plots, as appropriate. Chi-square test was used in the data analysis for categorical variables while Wilcoxon rank-sum test or Student’s t-test were used in the data analysis for continuous variables. Overall survival was studied using the Kaplan-Meier method and compared across groups using the log-rank test. In the multivariable-adjusted data analysis, the logistic regression model was used to assess odds ratios for categorical outcome variables such as MACE (yes/no), while the Cox proportional-hazards model was used to assess the overall survival and hazard ratios for comparison between SARS-CoV-2 negative and SARS-CoV-2 positive among CVD and Cardioonc respectively, adjusting for patient baseline characteristics. Because death was a terminal event for other outcomes such as stroke, acute heart failure or MI and because there were more deaths in the sicker patient subgroups, the cumulative incidences from each component in MACE including stroke, acute heart failure and MI were estimated and compared between different disease groups using competing risk analysis adjusting for the death – the terminal event. All p-values presented are 2-sided, and a p-value < 0.05 implies the statistical significance of this study. All analyses were conducted using Statistical software R. The competing risk analysis was carried out with the function in the cmprsk package.

We also estimated the survival probability for the major cancer types (lung, breast, prostate, colorectal, and hematological) in the Cardioonc (+) patients. Kaplan-Meier curves were used to visualize the corresponding survival probability, and the log-rank test to test statistical differences in survival probability between SARS-CoV-2 −positive and SARS-CoV-2 −negative patients. We also visualized survival curves by SARS-CoV-2 infection strain/evolution, dividing cases into early SARS-CoV-2, alpha, delta, and omicron cases.

Per N3C policy, exact counts that are 20 or less were not reported to protect the privacy of individuals. All analyses were performed in the N3C Data Enclave on the Palantir platform.

## Results

After inclusion/exclusion criteria, 418, 306 met the inclusion criteria and were included in the analysis. For each of the 4 cohorts of those with CVD; 309,086 (74%) were COVID-19 negative and 41,985 (10%) were SARS-CoV-2 positive; among those with both active cancer and cardiovascular disease, 1,414 (0.3%) were COVID-19 positive and 65,821 (16%) were SARS-CoV-2negative. The socio demographic characteristics are presented in Table 1. Of those in the study cohort, 56% were 65 years and older, and 45% female, and 71% were White.

## Prevalence of MACE and Death

We examined the prevalence of death and MACE in each of the four cohorts, both overall and stratified by periods of time when various SARS-CoV-2 variants were dominant (Table 2). During the early period of SARS-CoV-2, overall prevalence of death or MACE was 15% and 44%, respectively. During the same period, the rates of death and MACE were highest among Cardioonc (+) patients (Death, 38%; MACE, 55%), followed by Cardioonc (−) patients (Death, 26%; MACE, 47%), then CVD (+) patients (Death, 23%; MACE, 47%) and CVD (−) patients (Death, 11%; MACE, 43%). During SARS-CoV-2 Alpha variant period, overall death rate was 10%, and MACE was 39%, respectively; Cardioonc (+) patients experienced more deaths (31%) or MACE (49%). Among Cardioonc (−) patients, death rate was 17% and MACE was 37%. Among CVD (+) patients, death rate was 19%, MACE rate was 42%, finally among CVD (−) patients, death rate was 7% and MACE rate was 38%. When the Delta variant was prevalent, overall death or MACE rate was 8% and 36%. Similar to the previous variants, patients classified as Cardioonc (+) patients experienced the highest rate of death (23%) or MACE (41%). The rate of death or MACE was 14%, 33% among Cardioonc (−) patients; 17%, 37% among CVD (+) patients; and 6%, 36% among CVD (−) patients. Finally, during the Omicron variant period, with an overall death rate of 7% and MACE rate of 33%, Cardioonc (+) patients also had the highest rates of death (21%) or MACE (35%). Among Cardioonc (−) patients, death or MACE rate was 9% and 26%; among CVD (+) patients, death or MACE rate was 14% and 35%; and among CVD (−) patients, death or MACE rate was 4% and 34%.

## Odds of MACE

Compared to CVD (−) group, during the entire COVID-19 period, there was an overall increased odds of MACE among CVD (+) (OR = 1.14 [1.11–1.17]), Cardioonc (−) (OR = 1.33 [1.31–1.36]), and Cardioonc (+) patients (OR = 1.66 [1.47–1.87]) (Table 3). In the early SARS-CoV-2 period, CVD (+) (OR = 1.1.24 [1.20–1.28]), Cardioonc (−) (OR = 1.43 [1.40–1.46]), and Cardioonc (+) patients (OR = 1.93 [1.63–2.28]) patients exhibit higher odds of MACE compared to CVD (−) patients. During the Alpha variant period, compared to CVD (−) patients the odds of MACE was highest among Cardioonc (+) patients (OR = 1.86 [1.44–2.40]), then Cardioonc (−) patients (OR = 1.10 [1.04–1.16]), and finally CVD (+) patients (OR = 1.14 [1.08–1.20]). However, when the Delta variant was prevalent, there was no statistical difference in odds of MACE across all four groups.

## MACE events over time

**Figure 1:**
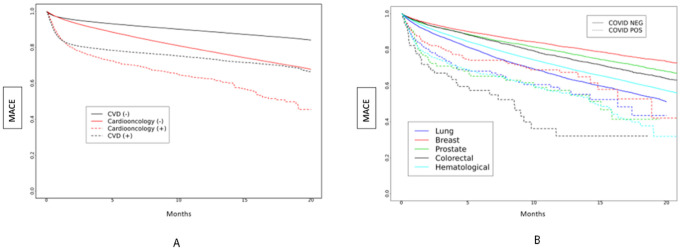
**A and 1B:** Major Adverse Cardiac Events (MACE) over time by study cohort (Figure 1A) and by cancer type (Figure 1B) over total study period

The Kaplan Meier curve of probability of MACE over time is presented in Figs. 1A–1B, showing significant differences between the four cohorts. Cardioonc (+) patients had the highest risk of MACE compared to the other groups (Fig. 1a). When we compared across cancer types, risk of MACE events was highest among persons who had colorectal cancer and had SARS-CoV-2.

## Subgroup Analysis

**Figure 2:**
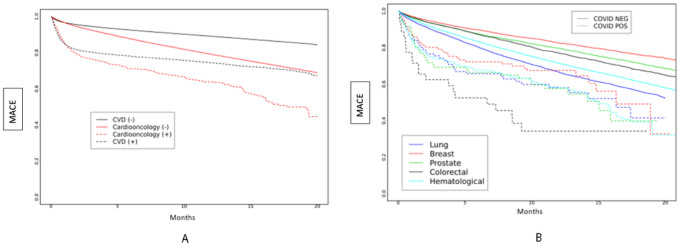
**A and 2B:** Major Adverse Cardiac Events (MACE) over time by study cohort (Figure 2A) and cancer type (Figure 2B) during the early COVID-19 period

**Figure 3:**
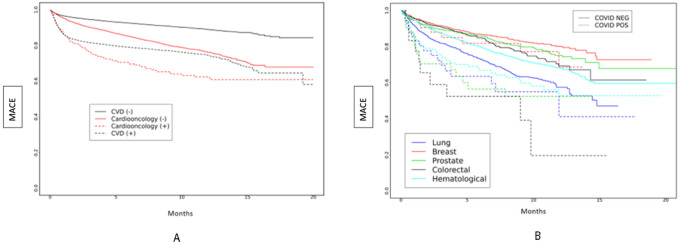
**A and 3B:** Major Adverse Cardiac Events (MACE) over time by study cohort (Figure 3A) and cancer type (Figure 3B) during the Alpha period

**Figure 4:**
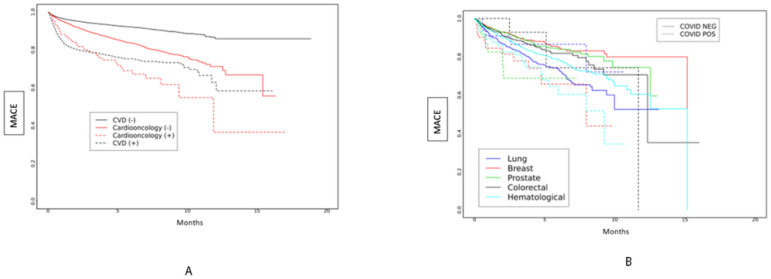
**A and B:** Major Adverse Cardiac Events (MACE) over time by study cohort (Figure 4A) and cancer type (Figure 4B) during the Delta period

**Figure 5:**
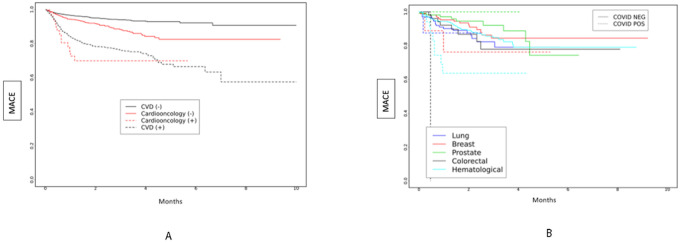
**A and 5B:** Major Adverse Cardiac Events (MACE) over time by study cohort (Figure 5A) and cancer type (Figure 5B) during the Omicron period

We examined the risk of MACE in subgroups based on periods of dominant SARS-CoV-2 variants in a survival analysis, which showed differences between groups (Figs. 2a–2b). During the early SARS-CoV-2 period, the risk of MACE was highest among Cardioonc (+) patients (Fig. 2a). Across all SARS-CoV-2 variant periods, there were differences in risk of MACE; in the early SARS-CoV-2 and Delta variant periods, risk of MACE was highest among Cardioonc (+) patients (Fig. 3–5).

## Competing Risk Analysis

As shown in Table 4, When we accounted for death as a competing risk on MACE with CVD (−) as a reference, we found that the patients in the Cardioonc (−) cohort had reduced HRs for stroke (HR:0.79; p < 0.001), acute heart failure (HR:0.76; p < 0.001), and myocardial infarction (HR:0.73; p < 0.001). In the Cardioonc (+) cohort, HR was not significant after including death as a competing risk for all three MACE components. In the CVD (+) cohort, had a higher hazard ratio for myocardial infarction (HR: 1.06; p < 0.001) despite accounting for deaths in the cohort.

## Discussion

The aim of our study was to report MACE outcomes in patients who had a cardiovascular diseases diagnosis with or without concomitant active cancers and, with or without documented SARS-CoV-2 infection. The N3C database, a large aggregate of patient data from 72 hospital EMRs across the US, provided a reliable and valuable dataset to address the knowledge gap. These results suggest that the SARS-CoV-2 infection, irrespective of cancer status, was associated with a higher rate of MACE; however, patients with active cancer with underlying cardiovascular conditions who were hospitalized with acute SARS-CoV-2 infection had poorer outcomes. The outcomes were significantly different amongst the groups earlier in pandemic, but there was no significant difference by cancer or CVD status in the later phases of pandemic (Central Illustration). When we looked at the most common cancer types, all cancers in the early course had similar probabilities for the events, but the patients with colon cancer had the worse intermediate term outcomes.

A study published by Ganantra and colleagues reported that the cardiooncology patients with SARS-CoV-2 had poorer outcomes than those with COVID-19 infection but did not have concomitant cancer and heart diseases.^5^ They observed that patients with active cancer and CVD are at elevated risk of MACE. However, the study included only four centers from Massachusetts, USA, and only included patients in the early phase of the pandemic. In our study, our data suggest similar results for patients admitted to the hospital in the early pandemic, while MACE outcomes were not significantly different for later phases of the pandemic (Table 3). The lack of knowledge about SARS-CoV-2 and its management, scarcity of hospital resources, training and availability of staff, the severity of viral strains, and unavailability of vaccinations are possible explanations for worse outcomes in the early pandemic.

Patients with active cancers are immunosuppressed, making them susceptible to infections given their attenuated immune response. Several studies involving patients with cancer and SARS-CoV-2 infection have shown that these patients are vulnerable to infections and poor outcomes.^8^ Earlier reports have shown that patients with CVD or CV risk factors have a higher case-fatality rate than those without CVD from SARS-CoV-2 infection.^9,10^ When we compared Cardioonc patients with or without SARS-CoV-2 infection, the presence of SARS-CoV-2 was associated with worse outcomes. The Cardioonc patients are even more prone to blunted immune response and multi-organ failure are less likely to hemodynamically compensate in the setting of active infection.^11^

When we considered death as a competing risk for stroke, acute heart failure and acute myocardial infarctions, the patients in the Cardioonc (−) cohort had a reduced risk for all three events. These findings suggest that death served as a competing risk for cardiovascular events from occurring, when compared patients who were in CVD (−) cohort. From our database, however, we cannot elucidate exact causes of death. CVD (+) patients had higher HR of myocardial infarctions, but reduced HR for stroke and acute heart failure events. In other words, death precluded the occurrences of stroke and acute heart failure in the CVD (+), while deaths precluded all three events were in the Cardioonc (−) cohort. In contrary, HR did not change significantly for Cardioonc (+) even after accounting for deaths. The most likely explanation is that the majority of patients in Cardioonc (+) cohort suffered deaths, and therefore did not experience other events. In all four cohorts, the events were mainly driven by deaths, which is an important highlight of our study.

At the beginning of the pandemic, early 2020, when nationwide shelter in pace, social distancing and other preventive measured were initiated, necessary cardiac procedures decreased significantly from pre-pandemic levels, which further influenced the outcomes in both cardio-oncology and CVD patients with active SARS-CoV-2 infections compared to their SARS-CoV-2 negative counterparts.^12^ As the pandemic progressed, easing restrictions for cardiac testing and procedures, availability of vaccination, and diminishing strength of SARS-CoV-2 viral strains may have eliminated the differences in outcomes by CVD or active cancer diagnosis in the later phases of the pandemic. The N3C database does not reliably capture the vaccine status of patients if the vaccines were administered outside of the healthcare systems. Therefore, we cannot draw any conclusion regarding the effectiveness of vaccinations in our study population. SARS-CoV-2 vaccines are highly effective in preventing severe illness in vaccinated subjects.^13–15^ Thus, the increasing SARS-CoV-2 vaccination over time likely influenced the SARS-CoV-2 outcomes later in the pandemic. A study reported by Tehrani and colleagues reported the findings that differ from our study, where the authors did not see increased risk of MACE in patients with cancer and cardiac conditions as compared to patients with cancer alone.^6^ Possible explanations for the discrepancies are the inclusion of patients with a history of cancer vs. active cancer status in our study, defined as patients with a cancer diagnosis and recent use of antineoplastic agent use; and the inclusion of all phases of pandemic in our study vs. Limited pandemic phase in the previous study.

We found that patients with active colorectal cancer at the time of infection had a higher cumulative probability of MACE than lung, breast, prostate, and hematologic malignancies. Interestingly, in the patients without acute SARS-CoV-2 infections, patients with lung cancers had a higher cumulative probability of MACE events. In a Veterans Affairs Healthcare System study, these authors reported poorer outcomes in patients with hematologic malignancies and infected with SARS-CoV-2 patients.^16^ In another study, liver and pancreatic cancers were associated with higher mortality rate.^17^ The exact mechanism behind the findings is unclear and may need further study. Regardless of cancer subtypes, the patients with cancer and active SARS-CoV-2 infection had higher cumulative probabilities of MACE in the acute phase of infection, and sustained at 20 month follow-up.

## Limitations

Despite the fact that N3C provides robust data to study SARS-CoV-2 infected patients, there are inherent limitations associated with administrative-style databases. The database does not provide severity of cardiovascular conditions. We cannot ascertain the details of metastatic and functional status from the information available in the database. We observed that there are significant discrepancies amongst the availability of variables, including laboratory and vital signs data that were missing for many organizations, and therefore, we chose to include or analyze incomplete and missing data. All authors agreed not to define a conditional based on laboratory or biomarker values (i.e., defining acute myocardial infarction based on troponin elevation), and restricted the case definition to the presence of specific diagnoses codes. Follow-up data outside of the participating institutes was not available which introduced bias related to missing follow-up information. Definitions of active cancer varies across literature; however, we restricted our cohort only to cancer therapy within 14 days of index hospitalization to ensure that there is no inclusion of patients in cancer remission and to capture people with active cancer treatment.

## Conclusion

Patients with acute SARS-CoV-2 infection in the early phase of pandemic suffered higher rates of MACE. The Cardioonc patients with active SARS-CoV-2 infection had the poorer outcomes. The effect of acute SARS-CoV-2 on MACE mitigated after early SARS-CoV-2 phase in all four cohorts.

## Figures and Tables

**Table 1:** Characteristics for all included patients

	CVD (−)	Cardioonc (−)	Cardioonc (+)	CVD (+)	Total	p-value*
	n(%)	n(%)	n(%)	n(%)	N(%)	
Patients	309086(74)	65821(15.7)	1414(0.3)	41985(10.0)	418306(100)	
Age < = 65	134225(46.7)	21471(34.3)	495(36.7)	14202(37.1)	170393(43.7)	< 0.001
Age > 65	153357(53.3)	41210(65.7)	852(63.3)	24029(62.9)	219448(56.3)	
Females	135466(43.8)	33503(50.9)	697(49.3)	18171(43.3)	187837(44.9)	< 0.001
White Race	213590(69.9)	50946(77.8)	1020(72.7)	26689(64.8)	292245(70.7)	
**Comorbidities**						
Alcohol Abuse	31569(10.2)	5866(8.9)	103(7.3)	2815(6.7)	40353(9.6)	< 0.001
Asthma	11059(3.6)	2616(4)	81(5.7)	1636(3.9)	15392(3.7)	< 0.001
Atrial fibrillation/Flutter	100962(32.7)	21392(32.5)	488(34.5)	14086(33.6)	136928(32.7)	0.001
Cerebrovascular Diseases	60334(19.5)	12024(18.3)	245(17.3)	6852(16.3)	79455(19.0)	< 0.001
Chronic Heart Failure	135556(43.9)	25021(38)	623(44.1)	19785(47.1)	180985(43.3)	0.001
Chronic Ischemic Heart Disease	205566(66.5)	42410(64.4)	853(60.3)	28195(67.2)	277024(66.2)	0.001
Chronic Kidney Disease	103117(33.4)	24505(37.2)	650(46)	17063(40.6)	145335(34.7)	0.001
COPD	24923(8.1)	8983(13.6)	204(14.4)	3506(8.4)	37616(9.0)	< 0.001
Diabetes	129406(41.9)	25523(38.8)	649(45.9)	22283(53.1)	177861(42.5)	< 0.001
Dyslipidemia	210353(68.1)	46153(70.1)	998(70.6)	28779(68.5)	286283(68.4)	< 0.001
History CABG	13188(4.3)	2286(3.5)	40(2.8)	1319(3.1)	16833(4.0)	< 0.001
Hypertension	234950(76.0)	53180(80.8)	1190(84.2)	31978(76.2)	321298(76.8)	< 0.001
Pacemaker ICD	2827(0.9)	733(1.1)	24(1.7)	409(1)	3993(1)	< 0.001
Peripheral Vascular Disease	214861(69.5)	45745(69.5)	945(66.8)	29209(69.6)	290760(69.5)	0.001
Prior Heart Transplant	3032(1)	867(1.3)	24(1.7)	350(0.8)	4273(1)	0.001
Prior Heart Valve	25645(8.3)	3730(5.7)	62(4.4)	1940(4.6)	31377(7.5)	0.001
Valvular Heart Disease	162957(52.7)	38163(58)	797(56.4)	16802(40)	218719(52.3)	0.001
**Antineoplastic agents**						
Cytotoxic agents	0(0)	19654(29.9)	791(55.9)	0(0)	20445(4.9)	< 0.001
Immunotherapy	0(0)	6525(9.9)	297(21)	0(0)	6822(1.6)	< 0.001
Endocrine therapy	0(0)	16839(25.6)	537(38)	0(0)	17376(4.2)	< 0.001
Targeted therapy	0(0)	9460(14.4)	382(27)	0(0)	9842(2.4)	< 0.001

Add Footnote: *Chi-square test with p-values was used to assess the proportions among four different cohorts.

CVD (−): Cardiovascular Disease and SARS-CoV-2 negative; Cardioonc (−): Cardiovascular Disease and Active Cancer and SARS-CoV-2 negative; Cardioonc (+): Cardiovascular Disease and Active Cancer and SARS-CoV-2 positive; CVD (+); Cardiovascular Disease and SARS-CoV-2 positive; COPD: chronic Obstructive Pulmonary Disease, CABG: Coronary Artery Bypass Graft, ICD:Implantable Cardioverter Defibrillator.

**Table 2:** Death and Major Adverse Cardiac Event (MACE) by group and viral variant

		CVD (−)	CVD (+)	Cardioonc (−)	Cardioonc (+)	Total	p-value*
Early SARS-CoV-2	Total cases	217282	22137	50212	737	290368	
	Death (%)	11.1	22.8	26.2	37.6	14.6	< 0.001
	MACE (%)	43.1	46.5	47.3	54.5	44.1	< 0.001
Alpha	Total cases	51579	9923	8870	324	70696	
	Death (%)	6.9	19.0	17.3	30.9	10.0	< 0.001
	MACE (%)	38.1	41.8	37.1	49.4	38.6	< 0.001
Delta	Total cases	34064	6440	5682	262	70696	
	Death (%)	5.5	16.7	13.8	22.5	8.2	< 0.001
	MACE (%)	36.2	37.0	33.0	40.8	36.0	< 0.001
Omicron	Total cases	6161	2166	1057	68	9452	
	Death (%)	3.7	14.0	8.9	20.6	6.8	< 0.001
	MACE (%)	33.8	34.9	25.5	35.3	33.2	< 0.001

* Chi-square test with p-values was used to assess the proportions among four different cohorts.

CVD (−): Cardiovascular Disease and SARS-CoV-2 negative; Cardioonc (−): Cardiovascular Disease and Active Cancer and SARS-CoV-2 negative; Cardioonc (+): Cardiovascular Disease and Active Cancer and SARS-CoV-2 positive; CVD (+); Cardiovascular Disease and SARS-CoV-2 positive

**Table 3:** Odds of Major Adverse Cardiac Event (MACE) from multivariable logistic models, adjusting for the baseline data including age, gender, BMI and other available patient characteristics.

Overall		
	OR (95% CI)	p-value
CVD (−)	Reference	
CVD (+)	1.14 (1.11–1.17)	< 0.001
Cardioonc (−)	1.33 (1.31–1.36)	< 0.001
Cardioonc (+)	1.66 (1.47–1.87)	< 0.001
Early SARS-CoV-2		
	OR (95% CI)	p-value
CVD (−)	Reference	
CVD (+)	1.24 (1.20–1.28)	< 0.001
Cardioonc (−)	1.43 (1.40–1.46)	< 0.001
Cardioonc (+)	1.93 (1.63–2.28)	< 0.001
Alpha		
	OR (95% CI)	p-value
CVD (−)	Reference	
CVD (+)	1.14 (1.08–1.20)	< 0.001
Cardioonc (−)	1.10 (1.04–1.16)	< 0.001
Cardioonc (+)	1.86 (1.44–2.40)	< 0.001
Delta		
	OR (95% CI)	p-value
CVD (−)	Reference	
CVD (+)	1.06 (0.99–1.13)	0.07
Cardioonc (−)	0.96 (0.90–1.03)	0.3
Cardioonc (+)	1.24 (0.93–1.65)	0.07
Omicron		
	OR (95% CI)	p-value
CVD (−)	Reference	
CVD (+)	0.99 (0.88–1.12)	0.96
Cardioonc (−)	0.71 (0.60–0.84)	< 0.001
Cardioonc (+)	0.89 (0.49–1.62)	0.71

CVD (−): Cardiovascular Disease and SARS-CoV-2 negative; Cardioonc (−): Cardiovascular Disease and Active Cancer and SARS-CoV-2 negative; Cardioonc (+): Cardiovascular Disease and Active Cancer and SARS-CoV-2 positive; CVD (+); Cardiovascular Disease and SARS-CoV-2 positive

**Table 4:** Hazard Ratios (HR) Accounting for Death as a Competing Risk from multivariable Cox models, adjusting for the baseline data including age, gender, BMI and other available patient characteristics.

Stroke	HR	p-value
CVD (−)	Reference	
CVD (+)	0.789	< 0.0001
Cardioonc (+)	0.901	0.2958
Cardioonc (−)	0.912	< 0.0001
**Acute Heart Failure**		
CVD (−)	Reference	
CVD (+)	0.7598	< 0.0001
Cardioonc (+)	0.6513	< 0.0001
Cardioonc (−)	0.813	<0.0001
**Myocardial Infarction**		
CVD (−)	Reference	
CVD (+)	0.7292	< 0.0001
Cardioonc (+)	0.9675	0.6659
Cardioonc (−)	1.0633	< 0.0001

CVD (−): Cardiovascular Disease and SARS-CoV-2 negative; Cardioonc (−): Cardiovascular Disease and Active Cancer and SARS-CoV-2 negative; Cardioonc (+): Cardiovascular Disease and Active Cancer and SARS-CoV-2 positive; CVD (+); Cardiovascular Disease and SARS-CoV-2 positive

## Data Availability

To access patient-level data from the N3C consortium, institutions must have a signed Data Use Agreement executed with NCATS and principal investigators must complete mandatory training along with submitting a Data Use Request (DUR) to N3C. All code used for analyses can be found on GitHub. To request N3C data access follow instructions at https://covid.cd2h.org/onboarding.
